# P266: Risk mapping the circuit of management of anaesthesia in obstetrics parturient to hospital Robert Debre

**DOI:** 10.1186/2047-2994-2-S1-P266

**Published:** 2013-06-20

**Authors:** M Adeoti, J Harthman, V Leroux, A Desroches

**Affiliations:** 1 Central Laboratory, CHU Yopougon, Côte d'Ivoire; 2 Hôpital Robert Debré, Paris, France; 3 Ecole centrale de Paris, Paris, Côte d'Ivoire

## Introduction

The optimal functioning of obstetric anesthesia circuit is a key element in the effectiveness of a hospital with a maternity activity since more than 80% of parturients benefits.

## Objectives

To analyze dangerous situations that may prevent obstetric anesthesia circuit to ensure anesthetic management of the parturient in optimum safety conditions of care.

## Methods

Based on the functional analysis, the preliminary risk analysis (PRA) is constructed in five steps: cutting functional circuit obstetric anesthesia, identification of dangerous events, hierarchy of feared events, ranking functions, establishment of recommendations to reduce or accept the risk.

## Results

The RPA has identified 93 hazardous situations and 178 scenarios which 266 criticality scenarios to criticality C_2_ and 88 to criticality C_3_. They must be action and risk control management of the residual risk for 24 of them. The main causes are related to preventable medical risk (error identification, error in anesthetic management), managerial risk (insufficient personal, non-compliance with instructions) and organizational risk (work overload, insufficient recycling). They are generally human error related to: patient characteristics (clinical status, homonymy, psychological state), the incompleteness of medical records (clinical summary, organization, transmission), the lack of protocol or protocols inadequate (obstetric anesthesia protocols), the failure of safety barriers (consultations and pre-anesthetic visit) and the difficulty of coordination exacerbated by local architecture and buildings.

## Conclusion

The use of this tool for risk management from industry can develop an approach to improving patient safety in obstetric anesthesia.

## Competing interests

None declared.

